# 

**Published:** 1843-07-01

**Authors:** 


### MEDICO-CHIRURGICAL

### REVIEW,

AND

### JOURNAL

OF

### PRACTICAL MEDICINE.

*(NEW SERIES.)*

VOLUME THIRTY-NINE,

[1st of APRIL, to 30th of SEPTEMBER,]

1843.

VOL. XIX. of DECENNIAL SERIES.

EDITED

By JAMES JOHNSON, M.D.

PHYSICIAN EXTRAORDINARY TO THE LATE KING,

AND OTHERS.

Surgeon Gen'l's Office LIBRARY 57479 Washington, D.C.

LONDON:

PUBLISHED BY S. HIGHLEY, 32, FLEET STREET,

*Re-printed in New York, by Mr. Wood.*

1843.

W1
ME 75 T

PRINTED BY F. HAYDEN,

Little College Street, Westminster.

### INDEX.

A.

Abscess, deep-seated, in the groin.... 177
Abscess in pelvic cellular tissue...... 223
Academician, droll request of a .... 525
Academy of medicine, memoirs of the 289
Acarus folliculorum............... 130
Acidulous alimentary principle...... 325
Acupuncture in neuralgia.......... 205
Affectation of would-be "Savans" .. 524
African remittent fever .......... 379
Agricultural chemistry, Liebig's .... 426
Agricultural chemistry, Johnston's .. 426
Agues, influence of the mind on .... 102
Agues, sulphate of iron in.......... 525
Air from the urinary passages ...... 276
Albuminous fluids, vegetable productions in ...................... 169
Albuminous urine, Bright & Barlow on 239
Aldridge on urinary diseases........ 271
Alexandria, health of the lazaretto at 175
Alimentary principles, composition of 311
Alimentary principles, classes of .... 321
Alimentary principles, aqueous...... 321
Alimentary principles, acidulous .... 325
Alimentary principles, proteinaceous 327
Alimentary principles, gelatinous .... 330
Alkalinity of the urine ............ 272
Alum, sale of, to bakers............ 566
American journal of dental science .. 150
Ammonia gas, inhalation of ........ 276
Ammoniacal urine, Bird on ........ 256
Amputation, on supra-malleolar .... 289
Amputation of the cervix uteri...... 454
Anatomy of strictures of the urethra 106
Andral on vegetable productions in albuminous fluids ................ 169
Andral on the carbonic acid of respiration .......................... 195
Anecdote of Decandolle............ 206
Animal magnetism in uterine affections 456
Animal magnetism in Paris ........ 496
Animalculæ in the blood............ 201
Antacids ........................ 351
Antagonism of certain diseases...... 518
Anthrax from carbuncle in cattle.... 176
Antidote of corrosive sublimate, proto-sulphuret of iron an.......... 178
Antilithics ...................... 351
Anti-neuralgic pills .............. 207
Antispasmodics .................. 31
Aphthæ of the cervix uteri ........ 452
Apoplexy, prognosis in .... ....... 189
Application of infants to the breast . 280
Aqueous alimentary principle ...... 321
Army, medical department of the .. 49
Arnal and Martin on amputations.... 289
Arsenic in squamous disease of the skin 126
Arsenic in inveterate syphilis ...... 526
Art of diving ..................... 178
Arteries and veins, morbid anatomy of 390
Ashwell on the diseases of women .. 357
Asthma, connexion of pulmonary emphysema with ................... 305
Astragalus, Turner on dislocations of 334
Auckland, temperature of the air at.. 567
Austria, Mr. Wilde's .............. 478

B.

Babington, character of Dr......... 17
Baillie, sagacity of ................ 202
Banner on paralysis of muscles...... 334
Begin on the arrest of hæmorrhage .. 291
Bell on the preparations of copaiva .. 551
Bird on ammoniacal urine.......... 256
Births, deaths, and marriages........ 397
Births, proportions of males to females 400
Bladder, tumors of the ............ 219
Bladder, distention of the .......... 219
Bladder, calculi in the ............ 220
Bladder, scirrhus of the............ 221
Blood, observations on the ......... 35
Blood, inferences drawn from coagulation of......................... 36
Blood, animalculæ in the .......... 201
Blood, Dr. Rees on the ............ 236
Blood, transmission of glanders by the, 523
Blood of dogs, entozoa in the ...... 527
Brain & spinal-cord, morbid anatomy of 394
Brain, state of the pupil in injuries of 551
Breast, application of infants to the.. 280
Bright & Barlow on albuminous urine, 239
Brighton, Dr. Wigan on ........... 93
Brilliant operation, decadence of a .. 524
Brodie, Sir B., dinner to .......... 573
Bull on the diseases of children .... 75
Bursæ, incision of ................ 246

C,

Cæsarian operation, successful case of 208
Cancer of the uterus .............. 368
Cantharides, extract of ............ 527
Capillaries action of the............ 140
Carbon of plants.................. 428
Carbon, on the ter-chloride of ...... 556
Carbonated waters, Venables on .... 474
Carbonic acid of respiration........ 195
Carbuncle in cattle, anthrax from... 176
Cardiac hypertrophy, relation of to renal disease.................. 240
Carpenter's physiology ............ 131
Catalogue of preparations, Langstaff's 384
Cathartics........................ 339
Cattle, anthrax from carbuncle in.... 176
Cauliflower excrescence of the uterus 374
Caustic, use of, in urethral diseases.. 106
Cephalotomy .................... 116

Cerebral symptoms from renal diseases 275
Cerebro-meningitis, epidemic, Rollet on 295
Cervix uteri, ulceration of the ...... 373
Cervix uteri, occlusion and rigidity of 374
Cervix uteri, aphthæ of the ........ 452
Cervix uteri, amputation of the .... 454
Chemical remedies ................ 346
Chemistry and microscopy, Simon on 35
Chemistry, agricultural, Liebig's .... 426
Chemistry, agricultural, Johnston's.. 426
Chevers on the causes of death after operations ..................... 225
Chevers on diseases of the coronary arteries ..................... 231
Child, food of the ................ 81
Children, Stewart on the diseases of.. 75
Children, Rees on the diseases of.... 75
Children, Bull on the management of 75
Children, Donné on the management of 75
Children, on the sleeping of ........ 84
Children, influence of air and exercise 84
Children, application of auscultation to 86
Children, sequelæ of scarlatina in... 88
Children, pneumonic and bronchitic attacks in ................. 89
Children, intestinal disorders in..... 90
Children, cholera of .............. 92
Children, Hocken on management of 280
Chomel on clinical observation...... 166
Chomel on the diagnosis of pneumonia 167
Churchill on strumous peritonitis.... 249
Civiale on strictures of the urethra .. 106
Civiale on the use of caustic in diseases of the urethra ............ 106
Clay on iodine as an injection ...... 552
Clendon on extraction of teeth...... 154
Climate of Brighton ............... 93
Climate of Van Dieman's Land...... 269
Cline, characteristics of ............ 3
Clinical observation, Chomel on, .... 166
Clinique chirugicale, Lisfranc's...... 445
Cod-liver oil in phthisis............. 503
Collard on temperaments .... ..... 291
Colles on diseases of the nail......... 260
Compliment to English genius ...... 207
Contagion of plague.............. 172
Cooper, Sir Astley, life of......... 1
Copaiva, Bell on the preparations of.. 551
Copper vessels, action of acids on .. 553
Coronary arteries, Dr. Chevers on diseases of the.................... 231
Corroding ulcer of the uterus ...... 374
Corrosive sublimate, antidote of .... 178
Cough, remedy for................ 553
Course of rheumatism in the horse .. 208
Creation, harmony of design through 528
Critical days, Recamier on.......... 198
Crowfoot on fractures of the spine .. 334
Cure of venereal warts ............ 281
Cynanche tonsillaris, Robertson's treatment of ................. 266

D.

Danish medical Journal, notice of a.. 522
Davy on M'Naughten's trial ........ 159
Death after operations, on the cause of 225
Death of Dr. Macleod ............ 282
Death of Mr. Tyrrel .............. 286
Death, registration of the causes of .. 408
Deaths, directions to the registrar of.. 278
Decadence of a brilliant operation .. 524
Decandolle, anecdote of............ 206
Delirium, pneumonia with.......... 200
Dental Science, American Journal of 150
Depletion in pericarditis............ 196
Design, harmony of, throughout creation ......................... 528
Devergie on the choice of a nurse.... 294
Diagnosis of pneumonia, Chomel on 167
Diagnosis of valvular disease of heart, 562
Diaphoretics, action of ............ 344
Digestion, organs of, morbid anatomy 395
Digestion and respiration .......... 506
Dinner to Sir B. Brodie ............ 573
Diphtherite, gingival .............. 195
Directions to the registrar of deaths.. 278
Diseases of the skin, Wilson on .... 119
Diseases of women, Ashwell on the.. 357
Diseases, geography of ............ 501
Dislocation of astragalus, Turner on 334
Diuretics ....................... 341
Diving, art of .................... 179
Dogs, entozoa in the blood of........ 527
Donné on the management of children 75
Downie on the waters of Heilbrunn.. 460
Droll request of an Academician .... 525
Dyspepsia ...................... 104

E.

Education versus marriage.......... 403
Electricity of steam, Faraday on the 550
Elements of pathology, Fletcher's .. 152
Elliotson on mesmerism............. 147
Emetics ........................ 337
Emmenagogues .................. 340
Emphysema of the lungs .......... 202
Emplastrum cerati saponis ....... .. 557
English genius, compliment to ...... 207
Entozoa in the blood of dogs ....... 527
Epidemic cerebro-meningitis, Rollet on 295
Epidemic miliary sweat, Parrot on .. 296
Epispastic, extract of cantharides as an 527
Erichsen on sensibility of the glottis.. 558
Examination of the medical boon.... 418
Expectorants, action of ............ 345
Extension in fractures of the spine . 334
Extract of cantharides, as an epispastic 527
Extraction of teeth, Clendon on .... 154

F.

Fallopian tube, tumors of the ...... 219

Fallopian tubes, diseases of the...... 456

False passage, in catheterism ....... 109

Faraday on the electricity of steam .. 550

Favus ......................... 129

Fecundity of females .............. 404
Fever, results of treatment in ...... 102
Fever, African remittent........... 379
Fistula ani, iodine as an injection in.. 552
Fletcher's elements of pathology .... 152
Food, Mélier on the supply of ...... 294
Food and diet, Pereira on ........... 307
Foods, chemical elements of ........ 308
Forensic medicine, Guy on ........ 462
Fork-grinding...................... 464
Formative hygiene, Royer-Collard on 297
Fractures of the spine, extension in.. 334
France, statistics of insanity in...... 500
French gratitude to medical men .... 206
French medical journal, profanity of, 527

G.

Galen, scraps from ................ 189
Geography of diseases ............. 501
Gibert on syphilitic eruptions ...... 299
Gigon on polypi of the rectum ...... 267
Gingival diptherite, case of .......... 195
Glanders in the human subject...... 233
Glanders, transmission of, by the blood 523
Glottis, on the sensibility of the .... 558
Gonorrhœa, treatment of ........... 206
Gonorrhœa, Child on the treatment of 555
Gout, Recamier and Tessier on...... 487
Gout and rheumatism, Todd on..... 475
Gratitude to medical men .......... 206
Groin, deep-seated abscess in the.... 177
Guy on forensic medicine .......... 462
Guy on the influence of the seasons.. 548
Guy's hospital reports ............. 209
Guy's Hospital .................... 282

H.

Hæmoptysis, Tessier on.......... .... 489
Hæmorrhage, Begin on the means of arresting ........................ 291
Hæmorrhoids, nitric acid in ....... 252
Hair, anatomy and physiology of the.. 122
Hamilton on painful affections of the nerves ...................... 257
Harmony of design throughout creation ......................... 528
Hastings on the water-cure ........ 545
Hastings on naphtha in phthisis .... 457
Health of the lazaretto at Alexandria, 175
Heart, malformations of the......... 385
Heart, morbid anatomy of the ...... 386
Heart, diagnosis of valvular disease of, 562
Heilbrunn, iodated waters of........ 460
Hemeralopia, oil of turpentine in .... 254
Herniæ, pelvic .................... 224
Hocken on the management of children 280
Holland's statistics of Sheffield ...... 463
Hooping-cough, treatment of ....... 103
Horse, course of rheumatism in the.. 208
Houston on nitric acid for hæmorrhoids 252
Hughes on glanders in the human subject ........................... 233
Human subject, glanders in the .... 233
Hydrocele, iodine injections in ...... 524
Hydrocele, spermatozoa in the fluid of 559
Hydrocephalus, Dr. Kennedy on .... 561
Hydropathic tour ................. 161
Hydro-pericardium, case of ........ 494
Hydrophobia ..................... 568
Hygiene, formative, Royer-Collard on 297
Hysteralgia ....................... 360
Hysteria ......................... 357

I.

Icthyosis sebacea ................ 128
Idiotcy in children, Voisin on ....... 151
Importance of veterinary medicine .. 206
Incision of bursæ .................. 246
Induction of premature labour ...... 365
Indurated tumors of walls of the uterus 363
Infants, application of, to the breast.. 280
Inflammation of the uterus .. ..... 366
Influence of the mind on agues...... 102
Inhalation of ammonia gas.......... 276
Insanity in France, statistics of...... 500
Insanity, Dr. Sutherland on ........ 529
Institute, successor of Larrey in the .. 523
Intermittent affections, Mélier on .... 301
Inveterate syphilis, arsenic in ....... 526
Iodated waters of Heilbrunn ........ 460
Iodine injections in hydrocele ....... 524
Iodine as an injection for fistula ani.. 552
Iron, proto-sulphuret of, an antidote of corrosive sublimate ........... 178
Iron, sulphate of, in agues.......... 525
Irritable uterus, Ashwell on the ..... 360
Irritant poisoning, case of .......... 213
Italian medicine, remarks on........ 497

J.

Jeffery on the topography of Sidmouth 333
Jeffreys on matico ................ 334
Johnson, Mr. H. J.'s report of cases 242, 537
Johnson, Mr. H. J. on ulcers of the legs 242
Johnson, Mr. H. J. on bursæ with melon-seed bodies .............. 246
Johnson, Dr. H., on mental disorders, 459
Johnston's agricultural chemistry.... 426

K.

Kennedy on scarlatina in Dublin .... 465
Kennedy, Dr., on hydrocephalus .... 561
Kidd on oil of turpentine in hemeralopia.......................... 254
Kidney, portal system of the........ 271
Kill or cure....................... 545
King, Dr., anecdote of ............ 14

L.

Langstaff's catalogue of preparations, 384
Larrey, successor of, in the Institute, 523
Laryngismus stridulus, pathology of.. 89
Lazaretto at Alexandria, health of the, 175
Leeches, how to make them bite .... 552
Legs, treatment of ulcers of the..... 242
Lever on pelvic tumors ............ 215
Liebig's organic chemistry.......... 426
Life of Sir Astley Cooper .......... 1

Life of a travelling physician......... 71
Light, influence of, on variolous pock, 124
Lisfranc's surgical clinique .......... 445
Lithic acid deposits................... 271
Livelihood, how to eke out a......... 207
Lungs, emphysema of ............... 202
M.
M'Cormack's methodus medendi .... 99
M'Cormac, Dr. on prolapsus ani .... 562
M'Cash on starched bandages ........ 555
Macleod, Dr., services of .......... 282
M'Naughten, trial of................. 159
M'William on the Niger expedition .. 377
Malformations of the heart......... 385
Malignant pustule, remarks on the .. 485
Marriages, age at which they take place......................399, 404
Marriages, Mr. Robertson on early .. 566
Marsh on strumous peritonitis ....... 251
Martin on supra-malleolar amputation 289
Materia medica, Simon's contributions to ........................ 467
Matico, remarks on ................ 334
Medical department of the army .... 49
Medical men, French gratitude to.... 206
Medical men, poverty of............ 207
Medical boon, examination of the.... 418
Medical topography of Sidmouth .... 333
Medical practitioners, notice to, .... 570
Medicinal substances, operations of.. 26
Medicinal substances, classification of, 28
Medicine, veterinary, importance of.. 206
Medicine, Memoirs of Royal Academy 289
Mélier on the supply of food......... 294
Mélier on intermittent affections .... 301
Mélier on the poisonous properties of quinine ......................... 306
Memoirs of Royal Acad. of Medicine.. 289
Memoranda on phlegmasia dolens.... 170
Mental disorders, Dr. H. Johnson on, 459
Mental derangement, Willis on...... 472
Menstruation, Raciborski on......... 510
Mesmerism, Elliotson on ........... 147
Methodus medendi, Dr. M'Cormack's, 99
Metritis, acute and chronic ......... 367
Microscopy and chemistry, Simon on, 35
Midwifery, Ramsbotham on ......... 112
Miliary sweat, epidemic, Parrot on .. 296
Military ophthalmia, origin of ...... 512
Milk, microscopical examination of the 294
Mind, influence of the, on agues .... 102
Moles ............................ 446
Morbid anatomy of the heart........ 386
Morbid anatomy of the pericardium.. 386
Morbid anatomy of arteries and veins, 390
Morbid anatomy of brain & spinal cord 394
Morbid phenomena, analysis of ..... 410
Morbus Brightii, state of the blood in, 236
Mortality, quarterly table of........ 278
Mortality in England ............... 400
Mortality, influence of the seasons on, 548
N.
Nail, Colles on diseases of the ....... 260
Nail, growing into the flesh .......... 260
Naphtha, treatment of phthisis by ... 457
Nerves, Hamilton on painful affections of the .......................... 257
Nervous system, elementary structure of the .......................... 133
Nervous coughing, how to stop...... 181
Neuralgia, acupuncture in........... 205
Niger expedition, M'William on the.. 377
Nitric acid in hæmorrhoids, Houston on 252
Nomenclature of disease............. 4[illegible]0
Nurse, Devergie on the choice of a .. 294
O.
Occlusion of the cervix uteri......... 375
Œsophagus, morbid anatomy of the.. 395
Oil of turpentine in hemeralopia .... 254
Onychia maligna.................... 263
Operations, Chevers on the causes of death after ...................... 225
Ophthalmia, military, origin of...... 512
Orfila on a new antidote for corrosive sublimate ...................... 178
Organic diseases of the uterine system, 361
Organic chemistry, Liebig's ........ 426
Os uteri, ulceration of the.......... 373
Ossification, history and progress of.. 462
Ovaries, tumors of the ............ 215
P.
Pain, on the utility of, ............ 526
Painful affections of nerves, Hamilton on 257
Palsy, Wilson on .................. 145
Paralysis of serratus magnus........ 334
Paris's pharmacologia .......... 24, 337
Paris, animal magnetism in ........ 496
Parrot on epidemic miliary sweat.... 296
Parturition, pelvic tumors obstructing, 215
Pathology, Fletcher's elements of.... 152
Pathology of phlegmasia dolens...... 481
Pau, climate of ................... 72
Pelvic tumors obstructing parturition 215
Pelvic cellular tissue, tumors of the.. 222
Pelvic abscess ..................... 223
Pelvic herniæ ..................... 224
Pereira on food and diet............. 307
Pereyra on cod liver oil in phthisis .. 503
Pericarditis, depletion in .......... 196
Pericardium, morbid anatomy of the.. 386
Perinæal herniæ .................... 225
Peritonitis, strumous, Churchill on .. 249
Pessaries, on the employment of...... 451
Pessary, new kind of ............... 279
Pharmacologia, Dr. Paris's ...... 24, 337
Phlebitis, occasional phenomenon in.. 191
Phlegmasia dolens, memoranda on .. 170
Phlegmasia dolens, pathology of .... 481
Phosphatic diathesis ............... 273
Phthisis, treated by naptha ........ 457
Phthisis, remarks on ............... 491
Phthisis, cod-liver oil in .. ....... 503

Physiology, Carpenter's.............. 131
Physiology, application of, to medicine 142
Physician, life of a travelling......... 71
Piorry on enlargement of the spleen.. 192
Placenta, general management of the 114
Plague, on the contagion of ........ 173
Plants, elements of composition of .. 428
Plants, mineral constituents of...... 438
Pleuritic effusion, cases of........ ... 492
Pneumonia, Chomel on the diagnosis of 167
Pneumonia with delirium .......... 200
Poisoning, irritant, case of.......... 213
Poisoning, on the different effects of 412
Poisonous properties of quinine...... 306
Polypi of the rectum, Gigon on...... 267
Polypi of the uterus .............. 445
Polypi of the vulva................ 446
Polypi of the vagina .............. 446
Population, progress of ............ 399
Population, analysis of the increase of 403
Portal system of the kidney ........ 271
Poverty of medical men.......... 207
Power on climate of Van Dieman's Land 270
Premature labour, on induction of .. 365
Preparations, Langstaff's catalogue of 384
Prescribing, Simon on the art of .... 467
Priessnitz, and hydropathy ........ 162
Principles of forensic medicine, Guy's 462
Profanity of a French medical journal 527
Professional success—gravity *v.* hilarity, 572
Prognosis in apoplexy ............ 189
Progress of population ............ 399
Prolapsus ani, Robert on the cure of.. 289
Prolapsus ani, Dr. M'Cormac on .... 562
Provincial association, transactions of 332
Prus on pulmonary emphysema .... 303
Puerperal convulsions ............ 117
Pulmonary emphysema ............ 185
Pulmonary emphysema, Prus on .... 303
Pupil, state of, in injuries of the brain 551
Purulent infection, Raciborski on.... 521

Q.

Quarterly table of mortality . ...... 278
Quinine, on the poisonous properties of 306
Quinine in rheumatism ............ 208

R

Raciborski on menstruation ........ 510
Raciborski on purulent infection .... 521
Ramsbotham on midwifery ........ 112
Rayer on typhus in lower animals.... 515
Read's, Mr. instruments............ 574
Recamier on critical days .......... 198
Recamier and Tessier on gout........ 487
Rectum, tumors of the ............ 219
Rectum, Gigon on polypi of the .... 267
Rees on the diseases of children .... 75
Rees on the blood in morbus brightii.. 236
Refrigerants...................... 349
Registrar of deaths, directions to .... 278
Registrar-general's report .......... 397
Registration of the causes of death .. 408
Renal disease a cause of death after operations ..................... 229
Renal disease and cardiac hypertrophy 240
Renal diseases, cerebral symptoms from 275
Report of med. department of the army 49
Respiration, carbonic acid of ...... 195
Respiration and digestion .......... 506
Respiratory movements at different ages 183
Rheumatism, course of, in the horse.. 208
Rheumatism, quinine in............ 208
Rigidity of the cervix uteri.......... 375
Robert on the cure of prolapsus ani .. 290
Robertson's treatment of cynanche tonsillaris ........................ 266
Robertson, Mr., on early marriages .. 566
Rollet on epidemic cerebro-meningitis 295
Royal Coll. of Surgeons—regulations of council ...................... 571
Royer-Collard on formative hygiene.. 297

S.

Sagacity of Dr. Baillie ............ 202
St. George's Hospital, reports from, 242 537
St. Thomas's Hospital ............ 281
Savans, affectation of would-be, .... 524
Scabies.......................... 126
Scarlatina in Dublin, Kennedy on.... 465
Scoutetten's hydropathic tour ...... 161
Scraps from Galen ................ 189
Seasons, influence of sickness........ 548
Sedatives, action of................ 30
Sedillot on a phenomenon in phlebitis 192
Serratus magnus, paralysis of the,.. .. 334
Sheffield, vital statistics of.......... 463
Sidmouth, medical topography of.... 333
Simon on chemistry and microscopy.. 35
Simon on the art of prescribing...... 467
Skin and urinary organs, connexion between ...................... 105
Skin, Wilson on diseases of the...... 119
Skin-diseases, classification of ...... 119
Skin, anatomy of the .............. 122
Skin, growth, &c. of the hair........ 122
Skin, congestive inflammation of the.. 123
Skin, arsenic in squamous diseases of 126
Skin, Willis on the special function of 255
Smee on the inhalation of ammonia gas 276
Snow's new kind of pessary ........ 279
Spasm, Wilson on ................ 144
Special function of the skin ........ 255
Speculum, on the employment of the, 362
Speculum oris, Dr. Holt Yates'...... 570
Spermatozoa in the fluid of hydrocele 559
Spine, extension in fractures of the .. 334
Spleen, connexion between enlargement of the, and intermittent fever .... 192
Spleen, Piorry or enlargement of the.. 192
Stammering, remarks on .......... 138
Starched bandage, M'Cash on the.... 555
Statistics of insanity in France ...... 500
Steam, Faraday on the electricity of, 550
Sterility, remarks on .............. 104

Stewart on the diseases of children .. 75
Stimulants, operations of .......... 28
Stomach, peculiar morbid affection of, 563
Stone, how to arrest hæmorrhage after operation for .................. 291
Stone, new solvent for ............ 553
Strictures, Civiale on............... 106
Strumous peritonitis, Churchill on .. 249
Strumous peritonitis, Marsh on....... 251
Suckling, diet during............... 79
Sulphate of iron in agues ........... 525
Supra-malleolar amputations ...... 289
Surgical clinique, Lisfranc's ........ 445
Sutherland, Dr. on insanity ........ 529
Syphilis, arsenic in inveterate ...... 526
Syphilitic eruptions, Gibert on ...... 299
T
Tea, on the use of ................. 526
Teeth, Clendon on extraction of....... 154
Temperaments, M. Collard on the .. 291
Ter-chloride of carbon, Tuson on the 556
Tessier on phlegmasia dolens ........ 170
Tessier on hæmoptysis ............ 489
Todd on gout and rheumatism ...... 475
Toe, Colles on affections of the nail of 260
Tooth-ache, remedy for ............ 552
Transactions of Provincial Association 332
Transmission of glanders by the blood 523
Travelling physician, life of a........ 71
Treatment of gonorrhœa ........... 206
Trial of M'Naughten ............... 159
Tumors of the ovaries............... 215
Tumors of the Fallopian tubes ...... 219
Tumors of the rectum ............ 219
Tumors of the bladder ............ 219
Tumors of the pelvic cellular tissue .. 222
Tumors of the walls of the uterus, .. 363
Turner on dislocation of the astragalus 334
Turpentine, oil of, in hemeralopia.... 254
Typhous fever, state of the urine in .. 274
Typhus in the lower animals, Rayer on 515
Tyrrell, Mr. death of ............... 286
U
Ulcer of the uterus corroding ...... 374
Ulceration of the os and cervix uteri.. 373
Ulcers of the legs, treatment of .... 242
Ure upon a new solvent for stone.... 553
Urethra, Civiale on strictures of the.. 106
Urethra, on the use of caustic in diseases of the .................. 106
Urinary organs and skin, connexion between ...................... 105
Urinary diseases, Aldridge on ...... 271
Urinary passages, air from the ...... 276
Urine, examination of the .......... 38
Urine, states of the, as a test of diseases 38
Urine, sediments of the ............ 40
Urine, alkalinity of the ............ 272
Urine in typhous fever ............ 274
Use of tea.......................... 526
Uteri, aphthæ of the cervix ........ 452
Uterine hæmorrhage .............. 116
Uterine system, organic diseases of the 361
Uterine tympanitis.................. 447
Uterine affections, magnetism in .... 456
Uterus, muscularity of the.......... 113
Uterus, rupture of the ............ 118
Uterus, Ashwell on the irritable...... 361
Uterus, indurated tumors of walls of, 363
Uterus, congestion and inflammation of 366
Uterus, cancer of the.............. 368
Uterus, corroding ulcer of the ...... 374
Uterus, cauliflower excrescence of the 374
Uterus, polypi of the .............. 445
Uterus, dropsy of the .............. 448
Uterus, inversion of the............ 448
Uterus, prolapsus of the .......... 449
Uterus, retroversion of the.......... 450
Uterus, anteversion of the.......... 450
Uterus, obliquity of the............ 451
Uterus, treatment of engorgements of 451
Uterus, non-cancerous ulcers of the.. 452
Uterus, cancer of the .............. 453
Utility of pain...................... 526
V.
Vaccination, theory of ............ 125
Vagina, polypi of the .............. 446
Vagina, expulsion of gas from the.... 448
Van Dieman's Land, climate of...... 269
Varicella an arrest of development of variola ........................ 125
Varices, Watson on ............... 559
Variolous pock, influence of light on 124
Vegetable productions in albuminous fluids ........................ 169
Vegetable chemistry ............... 426
Venables on carbonated waters ...... 474
Venereal warts, cure of ............ 281
Veterinary medicine, importance of.. 208
Vienna, state of disease in .......... 479
Vision, cause of erect.............. 136
Vital statistics of Sheffield .......... 463
Voisin on idiotcy in children........ 151
Vomiting, on the action of ........ 337
Vulva, polypi of the .............. 446
W.
Walls of uterus, indurated tumors of 363
Warts, venereal, cure of. ........... 281
Water as an alimentary principle .... 321
Water-cure followed by diseased heart 545
Watson on varices ................ 559
Westminster Hospital............... 281
Widows & orphans, society for relief of, 281
Wigan on Brighton.................. 93
Wilde's Austria .................. 478
Willis on the special function of the skin 255
Willis on mental derangement ...... 472
Wilson on diseases of the skin ...... 119
Wilson on spasm, languor and palsy.. 144
Women, Ashwell on the diseases of .. 357
Z.
Zoist, the.......................... 263

### INTELLIGENCE.

Dr. James Johnson has been elected a Corresponding Member of the "National Institute for the Promotion of Science" in Washington; for which he begs to return his best thanks through the Secretary, Francis Markoe, Esq.

### BIBLIOGRAPHICAL RECORD.

1. A Treatise on Mental Derangement. By Francis Willis, M.D. Fellow of the Royal College of Physicians. Longman & Co. 1843. Octavo, pp. 136.

2. A Lecture on Quack Medicines. Delivered before the Wakefield Mechanics' Institution, Feb. 1843. By T. G. Wright, M.D.

☞ *Very well adapted for its purpose.*

3. The Life of Sir Astley Cooper, Bart. interspersed with Sketches from his Note-Books of distinguished contemporary Characters. By Bransby Blake Cooper, Esq. F.R.S. In two vols, 8vo. J. W. Parker, 1843.

4. These sur Delirium Tremens. Par le Docteur Bougard, Chirurgeon de l'Hospice St. Gertrude, &c. Bruxelles, 1843.

5. Memoire sur l'Emploie du Carb. d'Ammoniaque dans la Scarlatine, &c. Par le Docteur Reiken, Medecin du Roi de Belge. Bruxelles, 1843.

6. A practical Treatise on the Diseases peculiar to Women; illustrated by Cases derived from Hospital and Private Practice. By Samuel Ashwell, M. D., Obstetric Physician to Guy's Hospital, &c. Part II. Organic Diseases. 8vo, pp. 226. Highley 1843.

7. Manual of British Botany, containing the Flowering Plants and Terms, arranged according to the Natural Orders. By Chas. C. Babington, F.L.S. &c. 8vo, pp. 400. Van Voorst, 1843.

8. General Therapeutics and Materia Medica, adapted for a Medical Text-book. By Robert Dunglison, M.D. Professor of Institutes of Medicine, &c. in Jefferson Medical College, Philadelphia. Two vols. 8vo. 1843. Philadelphia, Lea & Blanchard.

9. On certain Medical Delusions. An introductory Lecture. By Robert Dunglison, M.D. Philadelphia, 1843.

10. Discourse on the Objects and Importance of the National Institution for the Promotion of Science established at Washington. By Joel R. Poinset, Secretary at War, &c.

11. Report of the Pennsylvania Hospital for the Insane, for the Year 1842.

12. Bulletin of the Proceedings of the National Institution for the Promotion of Science, &c. Washington, 1841.
Second Bulletin do. 1842.

13. The Transactions of the Provincial Medical and Surgical Association. Instituted 1832. Vol. XI. Octavo. Churchill, London, 1843.

14. An Attempt to determine the Influence of the Seasons and Weather on Sickness and Mortality. By William August. Guy, M.B. Cantab., Professor of Forensic Medicine, King's College, London. Pp. 20. 1843.

15. Essays on partial Derangement of the Mind in supposed Connexion with Religion. By the late Dr. Cheyne. With a Portrait and Autobiographical Sketch. 8vo. W. Curry, Dublin—Longman and Co. London. 1843.

16. A History of British Birds. By W. Yarrell, F.L.S. Part 37—price 2s. 6d. Van Voorst, 1st June, 1843.

17. Mental Hygiene; or an Examination of the Intellect and Passions, &c. &c. By William Sweetster, M.D. late Professor, &c. New York, 1843. Wiley and Putnam, London. Octavo, pp. 270.

18. An Inaugural Lecture on Botany, considered as a Science, &c. By Edward Forbes, F.L.S. &c. Van Voorst, London, 1843.

19. The Art of Living. By Dr. Henry Duhring. Octavo, pp. 144. Longman & Co., June 1843.

20. Essays on Surgical Pathology and Practice. By Alex. Watson, M.D., F.R. C.S. E., Consulting Surgeon to the Royal Infirmary of Edinburgh, &c. Parts I. & II.

No. 1, on Abdominal Herniæ—2, on Injuries and Diseases of Arteries. Quarto, with Plates, 2s. 6d. per Number. Maclachlan and Stewart, 1843.

---

21. The British Quarterly Journal of Dental Surgery. Edited by J. ROBINSON, Esq. No. 1, Vol. I., March 1843. Churchill, London. 8vo, price 3s.

---

22. Guy's Hospital Reports. *Second Series.* No. 1, April 1, 1843. Conducted by Messrs. BARLOW, COCK, BIRKETT, BROWNE, and POLAND. S. Highley, Fleet Street, 1843. Price 6s.

---

23. Lectures on the Eruptive Fevers. Delivered at St. Thomas's Hospital in Jan. 1843. By GEORGE GREGORY, M.D. Fellow of the Royal College of Physicians, &c. &c. Octavo, pp. 258. Renshaw, 1843.

---

24. On Feigned and Factitious Diseases, chiefly of Soldiers and Seamen, &c. &c. By HECTOR GAVIN, M.D. Surgeon of the London Orphan Asylum, &c. Octavo, pp. 435. Churchill, London, 1843.

---

25. A Register of Experiments, Anatomical, Physiological, and Pathological, performed on Living Animals; disclosing New Views of the Circulation of the Blood in Man and Quadrupeds; with an Exposition of some Fallacies in the Harveian Doctrine. By JAMES TURNER, Veterinary Surgeon. Part II. pp. 100. Longman & Co. 1843.

---

26. Brighton, and its Three Climates; with Remarks on its Medical Topography, &c. By A. L. WIGAN, M.D. formerly practising in Brighton. 8vo, pp. 71. Highley, Fleet Street, 1843.

---

27 On the Amendment of the Law of Lunacy. A Letter to Lord Brougham. By a Phrenologist. Octavo, pp. 39. Renshaw, London, 1843.

---

28. An Essay on the Use of Nitric Acid as an Escharotic in certain Forms of Hæmorrhoidal Affection. Illustrated by Cases. By JOHN HOUSTON, M.D. 1843.

---

29. The Baths of Germany, considered with reference to their Remedial Efficacy, &c.; with an Appendix on the "Cold Water Cure." By EDWIN LEE, Esq. Second Edition, 1843. Whitaker and Co.

---

30. On Spasm, Languor, Palsy, and other Disorders, termed Nervous, of the Muscular System. By JAMES ARTHUR WILSON, M.D. Physician to St. George's Hospital. Octavo, pp. 200. Parker, 1843.

---

31. On the Anatomy of the Urinary and Sexual Organs, &c. By G. J. GUTHRIE, F.R.S. Surgeon to the Westminster Hospital, &c. Octavo, pp. 135. Third Edition. Churchill, 1843.

---

32. A practical Treatise on the Diseases of the Testis, and of the Spermatic Cord and Scrotum; with Illustrations. By T. B. CURLING, Lecturer on Surgery, &c. &c. Octavo, pp. 538. Longman and Co., May 1843.

---

33. Practical Remarks on Gout, Rheumatic Fever, and Chronic Rheumatism of the Joints: being the Croonian Lectures for the present Year, delivered at the College of Physicians. By ROBERT BENTLY TODD, M.D. F.R.S. &c. Small octavo, pp. 215. Parker, London, 1843.

---

34. The Plea of Humanity and Common Sense against two Publications;—one written by a "Physician incognito," &c. and the other by Mr. Yearsley. By JAMES WRIGHT, Esq. Mag. Hall, Oxford. Sherwood and Co., May 1843.

---

35. Austria; its Literary, Scientific, and Medical Institutions; with Notes, &c. By W. R. WILDE, M.R.I.A., Licentiate of the Royal College of Physicians in Ireland. Octavo, pp. 325. Curry and Co. Dublin, 1843.

---

36. The Educational and Subsidiary Provisions of the Royal Birmingham School of Medicine and Surgery. By the Rev. VAUGHAN THOMAS, B.D. (For private distribution).

---

37. A Treatise on Food and Diet; with Observations on the Dietetic Regimen suited for disordered States of the Digestive Organs, &c. By JONATHAN PEREIRA, M.D. F.R.S. &c. Octavo, pp. 542. Longman and Co., June 1843.

